# Aldose Reductase Inhibition Prevents Allergic Airway Remodeling through PI3K/AKT/GSK3β Pathway in Mice

**DOI:** 10.1371/journal.pone.0057442

**Published:** 2013-02-27

**Authors:** Umesh C. S. Yadav, Amarjit S. Naura, Leopoldo Aguilera-Aguirre, Istvan Boldogh, Hamid A. Boulares, William J. Calhoun, Kota V. Ramana, Satish K. Srivastava

**Affiliations:** 1 Department of Biochemistry and Molecular Biology, University of Texas Medical Branch, Galveston, Texas, United States of America; 2 Department of Microbiology and Immunology, University of Texas Medical Branch, Galveston, Texas, United States of America; 3 Department of Pharmacology and Experimental Therapeutics and Stanley Scot Cancer Center, Louisiana State University Health Sciences Center, New Orleans, Louisiana, United States of America; 4 Department of Medicine and Stanley Scot Cancer Center, Louisiana State University Health Sciences Center, New Orleans, Louisiana, United States of America; 5 Department of Internal Medicine-Pulmonary/Critical Care, University of Texas Medical Branch, Galveston, Texas, United States of America; French National Centre for Scientific Research, France

## Abstract

**Background:**

Long-term and unresolved airway inflammation and airway remodeling, characteristic features of chronic asthma, if not treated could lead to permanent structural changes in the airways. Aldose reductase (AR), an aldo-sugar and lipid aldehyde metabolizing enzyme, mediates allergen-induced airway inflammation in mice, but its role in the airway remodeling is not known. In the present study, we have examined the role of AR on airway remodeling using ovalbumin (OVA)-induced chronic asthma mouse model and cultured human primary airway epithelial cells (SAECs) and mouse lung fibroblasts (mLFs).

**Methods:**

Airway remodeling in chronic asthma model was established in mice sensitized and challenged twice a week with OVA for 6 weeks. AR inhibitor, fidarestat, was administered orally in drinking water after first challenge. Inflammatory cells infiltration in the lungs and goblet cell metaplasia, airway thickening, collagen deposition and airway hyper-responsiveness (AHR) in response to increasing doses of methacholine were assessed. The TGFβ1-induced epithelial-mesenchymal transition (EMT) in SAECs and changes in mLFs were examined to investigate AR-mediated molecular mechanism(s) of airway remodeling.

**Results:**

In the OVA-exposed mice for 6 wks inflammatory cells infiltration, levels of inflammatory cytokines and chemokines, goblet cell metaplasia, collagen deposition and AHR were significantly decreased by treatment with AR inhibitor, fidarestat. Further, inhibition of AR prevented TGFβ1-induced altered expression of E-cadherin, Vimentin, Occludin, and MMP-2 in SAECs, and alpha-smooth muscle actin and fibronectin in mLFs. Further, in SAECs, AR inhibition prevented TGFβ1- induced activation of PI3K/AKT/GSK3β pathway but not the phosphorylation of Smad2/3.

**Conclusion:**

Our results demonstrate that allergen-induced airway remodeling is mediated by AR and its inhibition blocks the progression of remodeling via inhibiting TGFβ1-induced Smad-independent and PI3K/AKT/GSK3β-dependent pathway.

## Introduction

Airway hyper-responsiveness (AHR) in asthma, one of the most prevalent chronic diseases [1], has been linked with airway inflammation and remodeling [2]. Age-related rapid decline in lung function has been found to be related to airway remodeling in asthmatics [3]. Mucous cells metaplasia and mucus hyper-secretion, epithelial-to-mesenchymal transition (EMT), collagen deposition and thickening of basement membrane in the airway are major contributing factors associated with chronic asthma-related airway hyper-responsiveness (AHR) in asthma patients [4]–[6]. Although many studies suggest the role of uncontrolled chronic inflammation and free radicals in the mediation of airway remodeling, a clear mechanism remains unknown [7], [8]. Further, airway remodeling leads to development of airway obstruction which occurs in many asthmatic patients with long-standing disease and present corticosteroid therapies are ineffective in preventing or treating this critical condition of asthma. We have shown previously that aldose reductase (AR) mediates early airway inflammatory response in ragweed pollen extract (RWE) and ovalbumin (OVA)-induced asthma and IL-13-induced mucous cell metaplasia [9]–[11]. However the role of AR in long term persisting airway inflammation leading to structural changes in the airways (remodeling) in chronic asthma is not known. We have already shown the efficacy of AR inhibitors in the allergen-induced acute airway inflammation, but prior to the clinical use of AR inhibitors in asthmatic patients to prevent or reverse airway inflammation and remodeling that leads to lung dysfunction, understanding the role of AR in airway remodeling and lung pathophysiology and the efficacy of AR inhibitors in such processes is necessary.

AR, a glucose metabolizing and regulatory enzyme of polyol pathway, is known to play a crucial role in the mediation of diabetic and cardiovascular complications [12]. Recently, several studies have suggested that AR mediates the pathophysiology of diseases unrelated to hyperglycemia, e.g. AR mediates LPS-induced acute lung and kidney injury, tumorigenesis and metastasis, periodontitis, mental disorders, and renal and ovarian abnormalities [13]–[20]. Further, increased expression of AR was observed in the lungs of chronic obstructive pulmonary diseases (COPD) patients [21]. These studies indicate that AR may be a key mediator in the airway remodeling in allergen-induced chronic inflammatory condition that leads to lung dysfunction. In this study, we have investigated the role of AR using a highly specific AR inhibitor, fidarestat, in controlling airway remodeling and dysfunction using a mouse model of OVA-induced lung inflammation. We have further examined the mechanism by which AR mediates TGFβ1-induced EMT and remodeling using cultured human primary small airway epithelial cells (SAECs) and primary mouse lung fibroblasts (mLFs). Our results demonstrate that inhibition of AR prevents airway remodeling in mice via regulating PI3K/AKT/GSK3β pathway.

## Methods

### Ethics Statement

All animal experiments were performed according to the National Institutes of Health Guide for Care and Use of Experimental Animals and approved by University of Texas Medical Branch Animal Care and Use Committee (Animal welfare assurance No. A3314-01).

### OVA-induced Asthma Model

**Figure 1:**
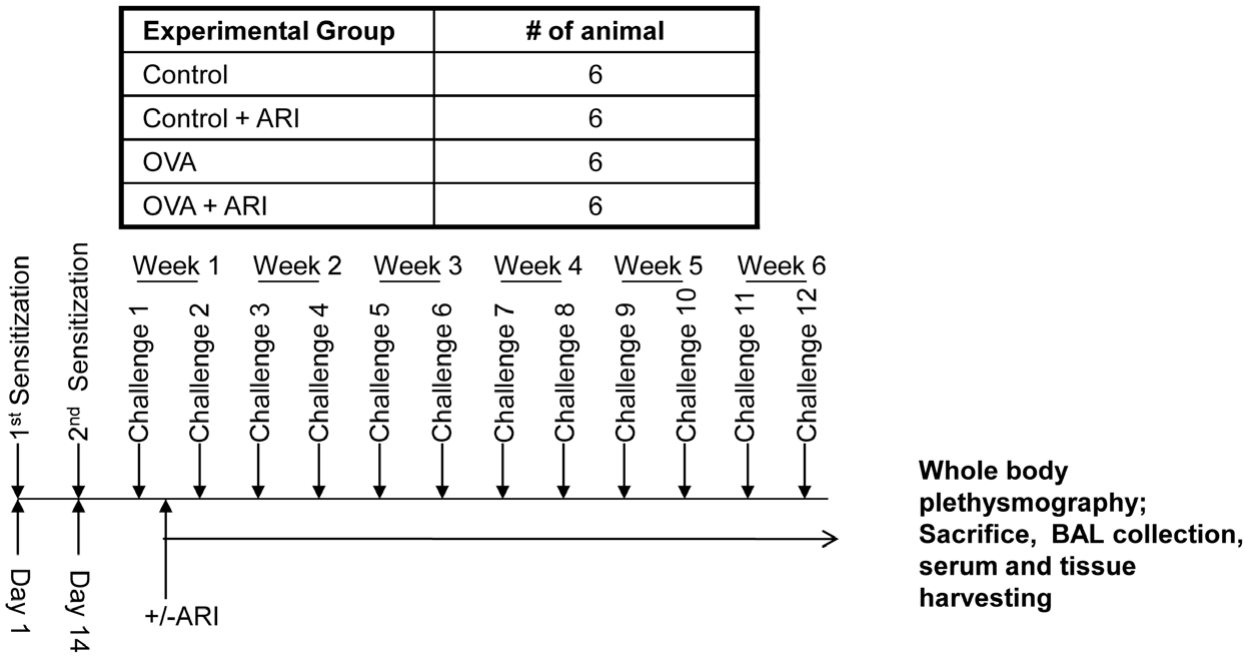
Experimental plan for chronic model of airway way inflammation and remodeling. Mice were sensitized and challenged with OVA for 6 weeks to induced airway inflammation and were treated with ARI starting after first OVA-challenge and continued till the end of experiment. At the end of experiments the mice were subjected to whole body plethysmography or were killed and serum and BAL fluid were collected. The lungs were perfused and fixed with 4% paraformaldehyde and harvested for histopathological studies (n = 6 per group) in two set of experiments. OVA, ovalbumin, ARI, aldose reductase inhibitor.

Six- to eight-weeks-old male (C57B/L6) mice were sensitized with 100 µg of grade V chicken OVA (Sigma-Aldrich, St. Louis, MO) mixed with 2 mg aluminum hydroxide in saline by *i. p.* injection once a week for two weeks as described [22]. Mice were then challenged with aerosolized 3% OVA for 30 min twice a week for 6 weeks as indicated in the Fig. 1 and were euthanized 48 h after the last challenge. The lungs were lavaged with 0.6 mL cold phosphate buffered saline (PBS) and BAL was processed for differential cell counting and determination of cytokines and chemokines as described below. In another set of experiments, the lungs were fixed with 4% paraformaldehyde and processed for histological examination after staining with H&E, PAS and Trichrome.

### Treatment with AR Inhibitor

The AR inhibitor, fidarestat (received as gift from Sanwa Kagaku Kenkyusho Co. Ltd, Japan, and Livwell, USA) was administered in drinking water provided ad-libitum such as that each mouse received ∼200 µg of the drug daily (calculated based upon milliliters of water consumption per day per mice). The treatment with ARI (10 mg/kg body weight) started after the first OVA challenge and continued until the mice were sacrificed.

### Assessment of Airway Hyper-responsiveness

Whole body plethysmography was performed to measure airway hyper-responsiveness in unrestrained and conscious mice 48 h after the last OVA-challenge. Enhanced pause (Penh) index values of airway hyper-reactivity were used as an indicator of changes in airway resistance. In brief, the baseline readings for 3 min were averaged after placing animal in a barometric chamber. Increasing concentrations of aerosolized methacholine were nebulized and readings were noted and averaged for 3 min after each nebulization and Penh values representing the airway hyper-responsiveness were calculated.

### Bronchoalveolar Lavage (BAL) Differential Cell Count

BAL samples were centrifuged at 800×g for 10 min and supernatants were frozen at −80C for assessment of inflammatory chemokines/cytokines. The cell pellets were resuspended in 250 µl of PBS containing 2% BSA, and the total cells were counted using automated counter (Coulter Electronics, Arlington, TX) and recorded as the total number of inflammatory cells per milliliter. The cell suspension was adjusted to a density of 20,000 cells per 100 µl and was cytospinned at 800×*g* for 10 min onto coated Superfrost Plus microscope slides (Baxter Diagnostics, Deerfield, IL). The cells on the slides were air-dried, and samples were stained with Diff Quick staining kit as described [10]. Differential cell counting was performed for ∼200 cells according to standard morphological criteria by a pathologist blinded to the treatment groups and the data presented as cells per milliliter.

### Cell Culture

The primary human small airway epithelial cells (SAECs) were obtained from Lonza (Walkerville, MD) and cultured according to the supplier’s instructions at 37°C in humidified incubator with 95% O_2_ and 5% CO_2_ in SABM as described [9], [10]. Cell passages 3–6 were used in the experiments. The primary mouse lung fibroblasts (mLFs) were isolated from age-matched naïve mice as described [23] and cultured in the growth medium containing 10% fetal bovine serum and 1% penicillin/streptomycin in Dulbecco’s modified Eagle’s medium (DMEM; Invitrogen, Carlsbad, CA) at 5% CO_2_ at 37°C. The mLFs were used at 80%confluence and passages 3–5. The cells were treated with 2 ng/ml of TGFβ1 (R&D Systems; Minneapolis, MN) at different time intervals as described in the respective figure legends.

### Western Blotting

After treatment, the cells were washed with ice-cold PBS and cell lysates were prepared in RIPA lysis buffer. The lysates were centrifuged at 12000 rpm for 10 min and equal amounts (40 µg) of proteins in the supernatants were subjected to electrophoresis on 10% SDS-PAGE. Subsequently, the proteins were electro-transferred to a nitrocellulose membrane, blocked with 5% nonfat milk in TBST, and probed with specific antibodies (all antibodies from Cell Signaling Technology; Danvers, MA) against various target proteins (1/1,000 dilution) and housekeeping proteins β-actin and GAPDH (1/10,000 dilution) for overnight at 4°C or for 2 h at room temperature. The blots were then washed, exposed to HRP-conjugated secondary Abs (1/5,000 dilutions) for 1 h at room temperature, and the Ag-Ab complex was detected by using Super Signal West Pico solution (Thermo scientific; Rockford, IL).

### Immunofluorescence Staining of Cells and Lung Sections

After the completion of incubation with TGFβ1, the cells were rinsed with PBS and fixed in 10% z-fix aqueous buffered-zinc formalin (Anatech Ltd; Battle Creek, MI) for 2 h at 4°C. The lungs were perfusion-fixed with 4% paraformaldehyde, embedded in paraffin, and cut into 5 µm sections. Antibodies against E-cadherin and vimentin in the SAECs and p-PI3K and p-GSK3β in the lung sections or isotope matched control IgG were used for immunofluorescent staining. Antigen specific binding of primary antibodies was detected using the Texas red- or FITC-labled secondary antibodies. The specimens were mounted with floursave mounting medium containing DAPI for nuclear staining. The slides were examined and microphotographed using a Nikon (EPI-800) epifluoroscence microscope.

### Statistical Analysis

Data presented as mean±SD with n = 6 for animals and n = 4 For cultured cells. and statistical significance was determined by unpaired t-test using graph pad prism software (GraphPad Software, Inc. La Jolla, CA). *p*<0.05 was considered as statistically significant.

## Results

### AR Inhibition Prevents Inflammatory Cells Infiltration in Chronic Asthmatic Mice Lungs

**Figure 2:**
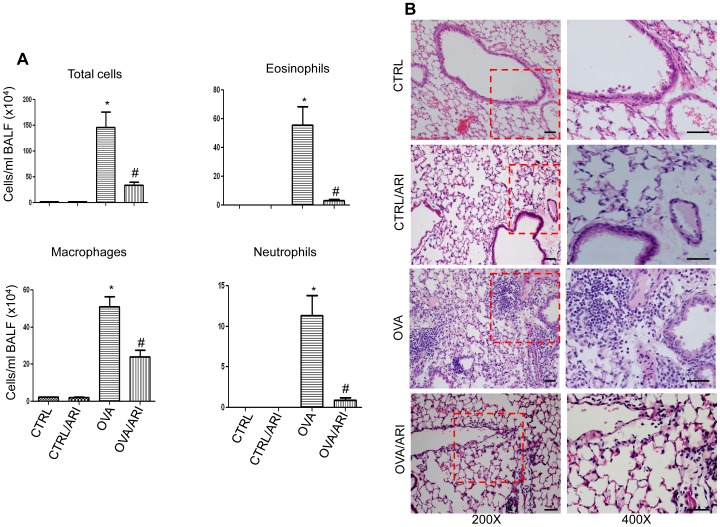
AR inhibition blocks leukocytes accumulation in BAL fluid and infiltration in the lungs of OVA–challenged chronic asthmatic mice. (A) The total as well as differential cell counts were performed in BAL fluids collected 48 h after final OVA-challenge. The total cells, eosinophils, neutrophils and macrophages per ml BAL fluid is given as mean ± SD (n = 6 per group), *p<0.01 vs control; ^#^ *p*<0.05 OVA group (B) Fixed lungs from the different experimental groups were sectioned and stained with hematoxylin and eosin, examined under the light microscope and photomicrographs were taken. A representative image from each group is shown (n = 6), Magnifications 200× and 400×. CTRL, control, ARI, aldose reductase inhibitor, OVA, ovalbumin.

During chronic asthma, antigen-induced airway inflammation is marked by increased number of inflammatory cells in the airways and in the lung sub-epithelial spaces [9], [10]. We therefore, examined the effect of AR inhibition on the infiltration of inflammatory cells in the lung and their accumulation in BAL fluid after multiple antigen challenges. As shown in Fig. 2A there was a significant increase in the number of total cells in the BAL fluid from the OVA-sensitized and -challenged mice for consecutive 6 weeks, whereas the number was significantly (∼75%) less in the BAL fluid of OVA-sensitized and -challenged mice treated with fidarestat. Further, the number of macrophages decreased significantly (>50%) while that of eosinophils and neutrophils decreased by 80–90% in the BAL fluid of fidarestat-treated OVA-challenged mice as compared to OVA-challenged but not treated with fidarestat. Similarly, increased inflammatory cells infiltration, particularly of eosinophils in the peri-vascular and peri-bronchial spaces of OVA-challenged mice shown in the Fig. 2B indicates persistent inflammation. On the contrary, in fidarestat-treated and OVA-challenged mice, infiltration of eosinophils in the lung tissue was markedly decreased when compared to OVA-challenged mice (Fig. 2B).

#### AR inhibition protects against airway hyper-responsiveness in chronic asthmatic mice

**Figure 3:**
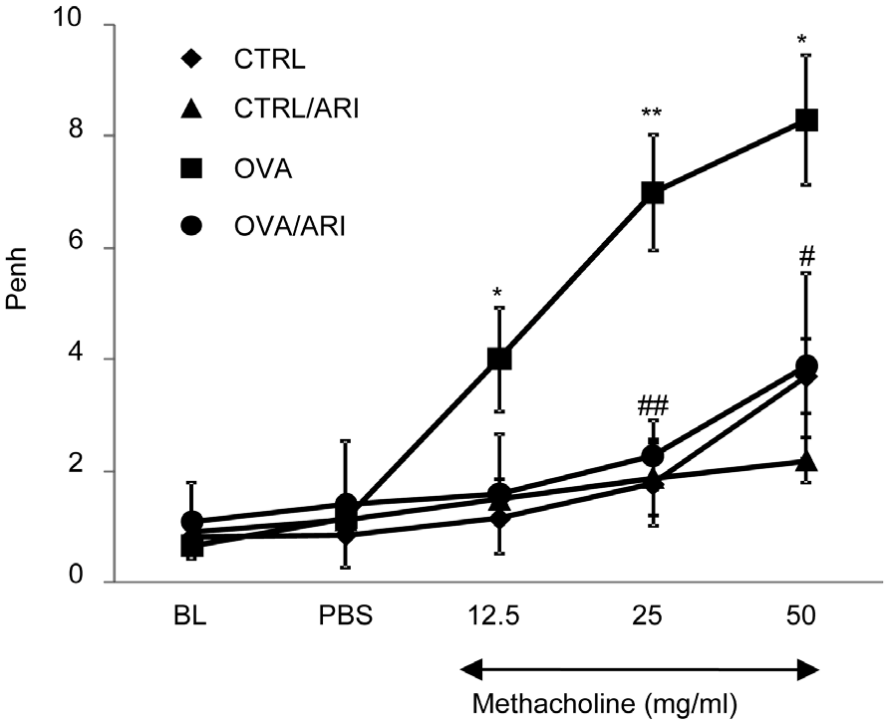
AR inhibition decreases airway hyper-responsiveness in the OVA-challenged chronic asthmatic mice. After 6-weeks of OVA-challenge, the changes in pause of breathing (Penh) were measured by whole-body unrestrained plethysmography using increasing concentration of methacholine. The mice from different experimental and control groups were placed in a barometric chambers and Penh values were determined. Each data point represents mean ± SD from each group (n = 4). *p<0.05 vs. control; **p<0.001 vs control; ^#^ *p<*0.01 vs OVA-challenged mice; BL, baseline; CTRL, control, ARI, aldose reductase inhibitor, OVA, ovalbumin, PBS, phosphate buffered saline.

Airway hyper-responsiveness (AHR) is an indicative of chronic inflammation in the airways, which results from the airflow restriction due to mucus hyper-secretion and remodeling leading to airway obstruction [24], [25]. Therefore, we measured AHR in the OVA-sensitized and -challenged (for 6 weeks) mice by whole body plethysmography and response to increasing doses of methacholine challenge was quantitatively determined. As shown in Fig. 3, in OVA-challenged mice there was a dose-dependent increase in the Penh values in response to methacholine challenge as compared to the control mice, which were not sensitized and challenged with the carrier (saline) alone. On the contrary, in fidarestat-treated mice sensitized and challenged with OVA for 6 weeks, the Penh values were significantly (∼70%; p<0.01) less when compared to OVA-sensitized and challenged mice but not treated with fidarestat. These results indicate that inhibition of AR significantly prevents airflow restriction caused by chronic allergic airway inflammation in mice.

### AR inhibition Protects against OVA-induced Mucous Cell Metaplasia and Mucus Hyper-secretion in the Mice Airways

**Figure 4:**
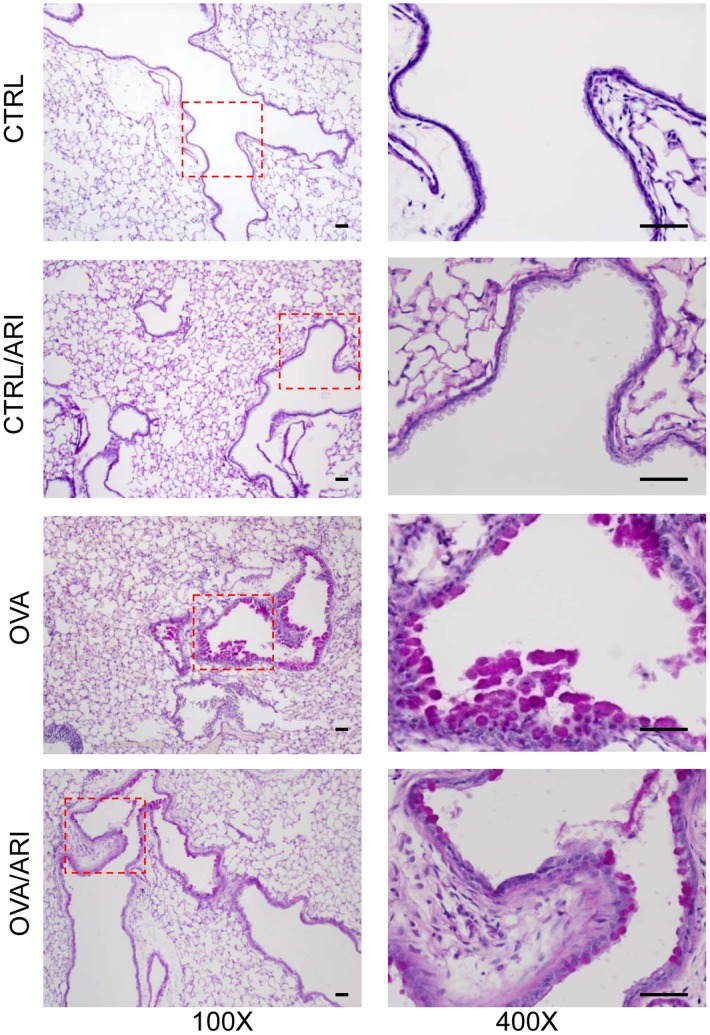
AR inhibition decreases OVA-induced metaplasia of mice lung epithelial cells. The lung sections of mice after 6-weeks of OVA-challenge were stained with PAS and observed under light microscope and photomicrographs were acquired. A representative photomicrograph is shown (n = 6). Magnifications 100× and 400×. CTRL, control, ARI, aldose reductase inhibitor, OVA, ovalbumin.

Since airway hyper-responsiveness has direct correlation with the excessive mucus secretion that is known to cause airway obstruction [26], [27], we determined the effects of AR inhibitor, fidarestat on mucous cell metaplasia and subsequent mucus hyper-secretion in the chronic allergic asthma mice. We first stained the mice lung sections with periodic acid Schiff (PAS) and determined the number of PAS positive cells. As shown in Fig. 4, increased numbers of PAS positive cells were observed in lung epithelium of OVA-sensitized and -challenged mice, whereas fidarestat-treated group showed markedly decreased number of PAS positive cells. These results indicate that AR inhibition effectively decreases airway metaplasia in response to long-term allergen exposure, which could be responsible for decreased AHR in OVA-challenged and fidarestat-treated mice.

### AR Inhibition Prevents Airway Remodeling in Chronic Asthmatic Mice

**Figure 5:**
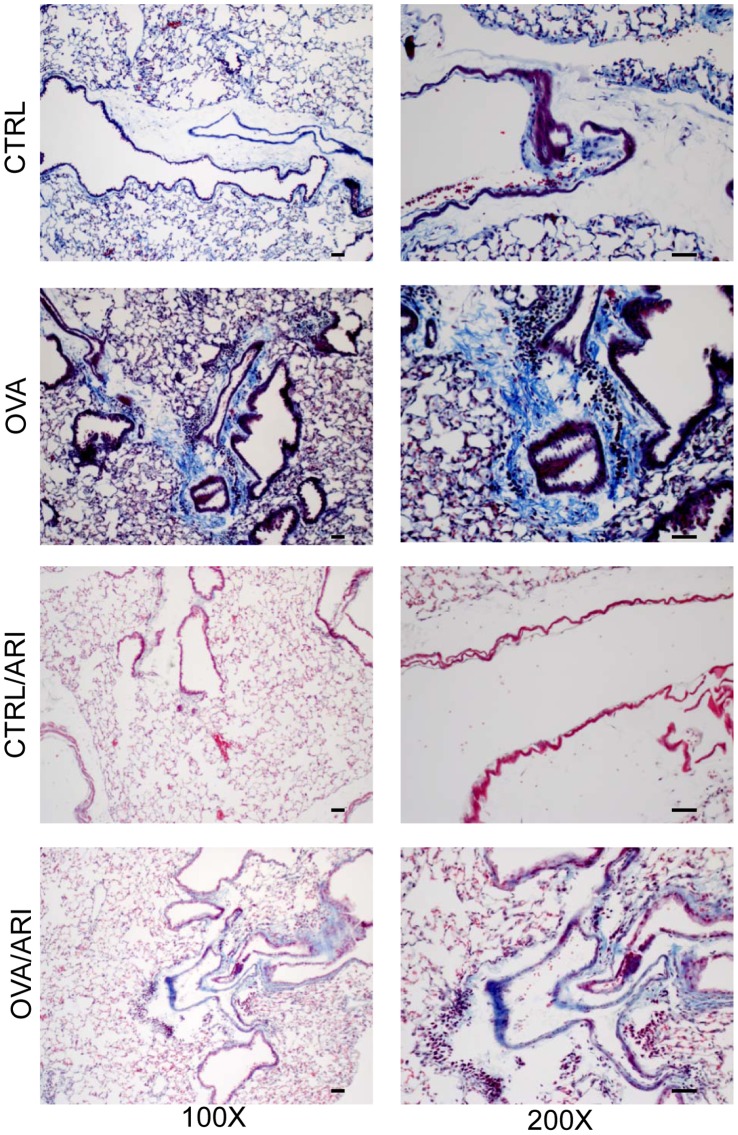
AR inhibition decreases airway remodeling in chronic asthmatic mice lungs -. The lung sections of mice after 6-weeks of OVA-challenge were stained with Masson’s trichrome staining and observed under light microscope and photomicrographs were acquired. A representative photomicrograph is shown (n = 6). Magnifications 100× and 200×. CTRL, control, ARI, aldose reductase inhibitor, OVA, ovalbumin.

Next, we examined the efficacy of orally administered fidarestat in the airway remodeling, a hallmark of chronic asthma. After 6 weeks of OVA-challenge, the mice lungs were harvested and Masson’s trichrome-staining was performed on the lung section. As shown in Fig. 5, characteristics of airway remodeling as determined by the deposition of collagen in the epithelial cell lining (blue color) and thickening of the basement layer below the epithelium in OVA-challenged group were observed. The decreased staining in the fidarestat-treated mice lungs corresponded with the significantly decreased airway remodeling (Fig. 5, lowest panel). These results correlate with the observation of increased AHR, mucous cell metaplasia and increase in inflammatory cytokines and chemokines (data not shown) and suggest that AR inhibition decreases airway inflammation and airway remodeling in a chronic model of allergic asthma in mice. In these experiments, the control mice treated without or with fidarestat alone did not show any remodeling.

### AR Inhibition in SAECs Prevented the Expression of TGFβ1-induced EMT Markers

**Figure 6:**
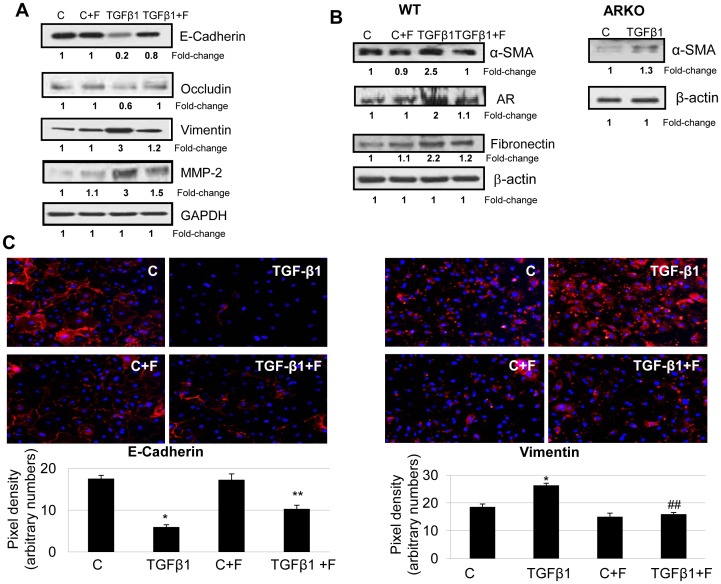
AR inhibition prevents TGFβ1-induced changes in EMT marker proteins in SAECs and mLFs. The (**A**) SAECs, and (**B**) mLFs derived from wild-type (WT) and AR-knockout (ARKO) mice were treated with TGFβ1 for 72 h, cell lysates were prepared and equal amounts of protein were analyzed for the expression of EMT marker proteins by Western blot analysis (n = 4). The numbers below the blots indicate fold-change in the band intensities. (**C**) The cells on chambered slides were fixed and immunostained with E-cadherin or vimentin specific antibodies using Texas red-labeled goat anti-rabbit secondary antibodies and mounted with floursave mounting medium containing DAPI. The photomicrographs were acquired by fluorescence microscopes. A representative field for each group is shown (magnification: 200×); (n = 4). The Bar diagrams below the photomicrographs indicate respective pixel densities showing relative changes in the expression. *p<0.01 Vs Control; **p<0.05 Vs TGFβ1 alone; ^##^p<0.01 Vs TGFβ1. C, control; F, fidarestat; TGFβ1, transforming growth factor-beta 1.

Since TGFβ1 is a well known mediator of airway remodeling in chronic asthma [28], we next investigated the effect of AR inhibition on TGFβ1-induced changes in the expression of structural proteins in SAECs and mLFs. Incubation of SAECs with TGFβ1 for 72 h without AR inhibitor caused significant decrease in the expression of epithelial cell marker proteins, E-cadherin (by ∼80%) and occludin (by ∼40%) while the expression of EMT marker proteins such as vimentin, and matrix metalloproteinase (MMP)-2 increased by approximately 3-folds. The treatment of SAECs with fidarestat reversed the expression pattern of these proteins (Fig. 6A). Similarly in mLFs, TGFβ1-induced increase in the expression of α–SMA and fibronectin was also significantly (by >90%) prevented by AR (Fig. 6B). Interestingly, TGFβ1 stimulation also increased the expression of AR in mLFs which reversed to near control levels by AR inhibitor treatment (Fig. 6B, left panel). Further, in mLFs derived from ARKO mouse lung, TGFβ1-induced expression of α-SMA was markedly lowered than that of WT mLFs (Fig. 6B, right panel). We next confirmed TGFβ1-induced changes in the expression of E-cadherin and vimentin in SAECs by immunofluorescent staining. As shown in Figure 6C (left panel), while cells in the control group showed E-cadherin expression on the cell membrane (red fluorescence), TGFβ1-treated SAECs showed markedly decreased expression of E-cadherin. On the other hand TGFβ1-treated SAECs showed increased expression of vimentin (Fig. 6C, right panel). Treatment with fidarestat significantly reversed the changed expression of both E-cadherin (by >50%; p<0.05) and vimentin (p<0.01) in SAECs as evident by respective quantitative pixel density shown below the microscopic images. These observations thus suggest that AR plays an important role in TGFβ1–induced changes in SAECs and mLFs.

### AR Inhibition in SAECs Prevented TGFβ1-induced EMT in Smad-independent Manner

**Figure 7:**
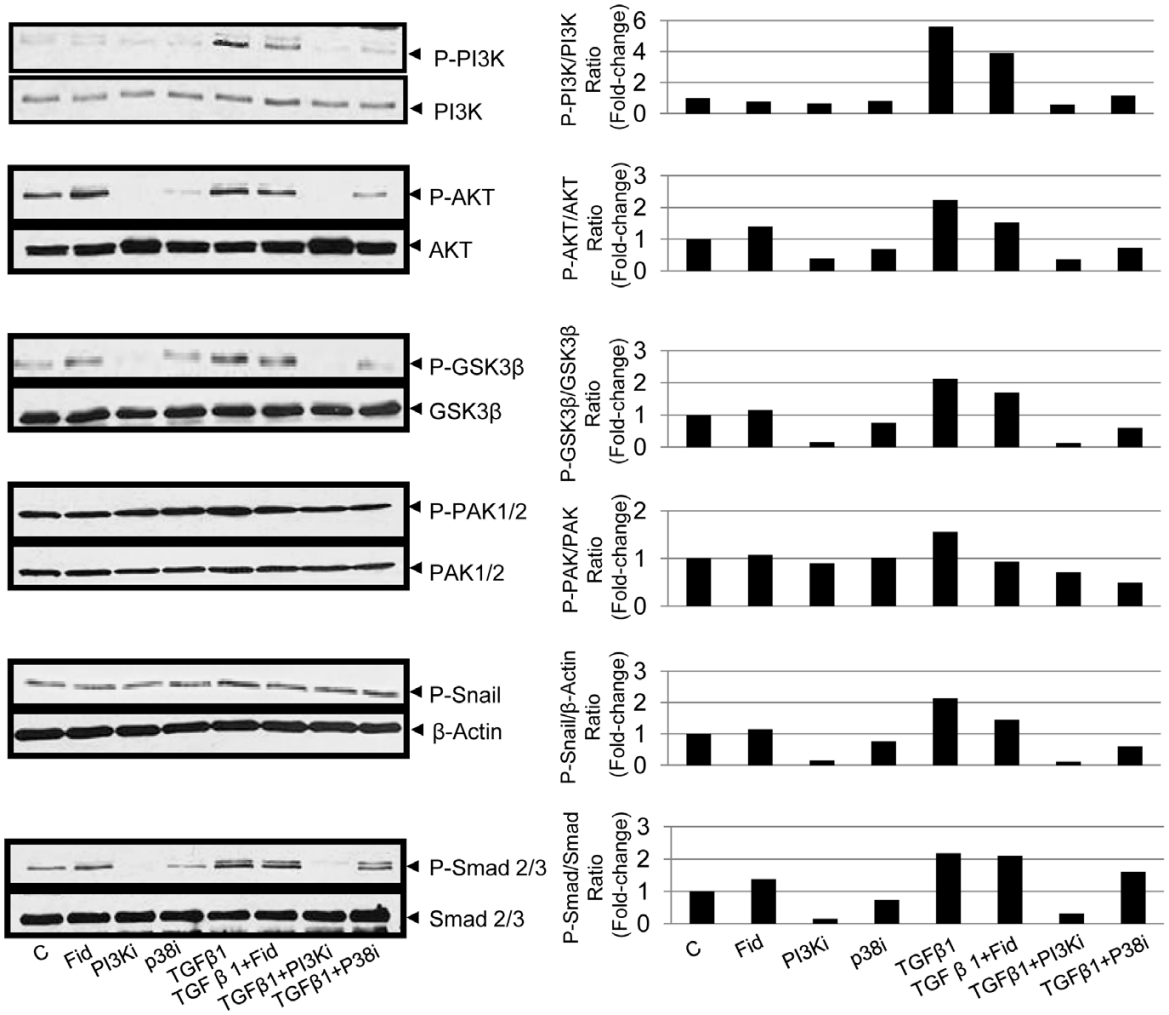
AR inhibition prevents TGFβ1-induced phosphorylation of PI3K/AKT and GSK3β and other signaling intermediates in SAECs. The SAECs were seeded in 6-well plate and starved for overnight with or without fidarestat and stimulated with TGFβ1 and incubated for 1 h. At the end of incubation period cells were washed with cold PBS and cell lysate was subjected to Western blotting using antibodies against phosphorylated-PI3K, AKT-1/2/3, GSK3β, PAK-1/2/, Snail and Smad2/3 to assess the activation of signaling kinases. A representative blot with respecitve densitometry data (bar diagram) is shown (n = 4). C, control, Fid, fidarestat, PI3Ki, PI3K inhibitor, TGFβ1, transforming growth factor-beta 1, P38i, P38 inhibitor.

We next examined the role of AR in TGFβ1-mediated airway remodeling in SAECs. TGFβ1-induced Smad2 signaling is one of the well known mechanisms that mediates airway remodeling in asthmatics [29]. We observed that although TGFβ1 induced a significant increase in the phosphorylation of Smad 2/3 in SAECs, AR inhibition did not prevent it. Since a number of studies implicate the role of TGFβ1-induced Smad-independent protein kinases such as PI3K in the airway remodeling [30], [31], we examined the effect of AR inhibition on the Smad-independent kinases in SAECs. TGFβ1-treatment caused increase in the phosphorylation of AKT, GSK3β and PAK protein kinases, which are known to play a significant role in EMT, were markedly decreased by inhibition of AR. On the other hand, PI3K inhibitor and P38 inhibitor almost completely blocked the phosphorylation of these kinases (Fig. 7). Since AR inhibition markedly prevented the phosphorylation of PI3K, AKT, GSK3β and PAK, and also the activation of Snail in Smad-independent manner, which is known to regulate E-cadherin expression, our results suggest that AR mediates TGFβ1-induced EMT and cellular changes in airway epithelial cells.

### AR Inhibition Prevented PI3K and GSK3β Phosphorylation in the Lungs of OVA-challenged Mice

**Figure 8:**
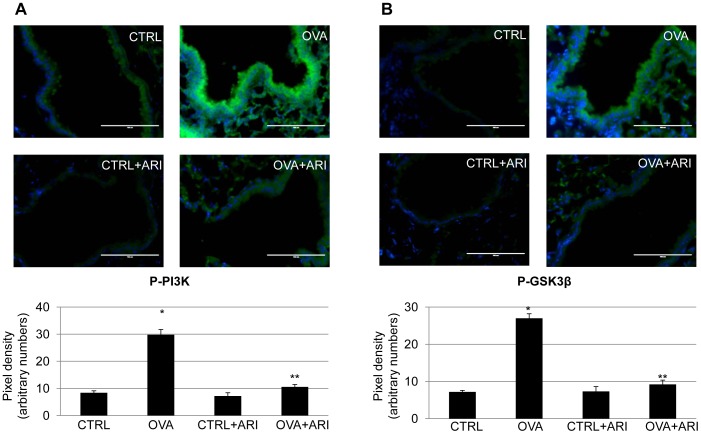
AR inhibition prevents phosphorylation of PI3K and GSK3β in OVA-challenged mouse lung epithelium. The lung sections were immunostained with (**A**) P-PI3K and (**B**) **P-**GSK3β specific antibodies using FITC-labeled goat anti-rabbit secondary antibodies and mounted with floursave mounting medium containing DAPI. The isotype matched control showed no IgG-specific fluorescence (not shown). Photomicrographs were acquired by fluorescence microscope. A representative field for each group is shown (magnification: 400×); (n = 4). The Bar diagrams below the photomicrographs indicate respective pixel densities showing relative changes in the expression. *p<0.01 Vs CTRL; **p<0.01 Vs OVA alone. CTRL, Control; OVA, ovalbumin; ARI, aldose reductase inhibitor.

To further confirm that TGFβ1-induced changes in SAECs are blocked by AR inhibition, we next examined the effect of AR inhibition on the phosphorylation of PI3K and GSK3β in the OVA-challenged mice lungs by immunohistochemistry using specific antibodies. As shown in Fig. 8, there was a marked increase in the fluorescence intensity specific to phospho-PI3K (Fig. 8A) and GSK3β (Fig. 8B) in the lung epithelium, while the lungs of control mice showed minimal staining. The fidarestat-treated mice showed a significantly (>90%) decreased fluorescent intensity as indicated by quantitative values of pixel densities (below the respective microscopic images) suggesting inhibition of phosphorylation of PI3K and GSK3β by AR inhibitor. These results suggest that inhibition of AR could prevent the activation of PI3K and GSK3β in OVA-challenged mice lungs and thereby prevent airway epithelial remodeling.

## Discussion

Our previous studies demonstrate that inhibition of AR prevents allergen-induced airway inflammation and Th2 response in mice caused by short-term exposure to RWE and OVA in sensitized animals [9], [10]. Although we have observed increased AR expression in the lungsections from the severe asthma patients (unpublished observation), the role of AR in airway remodeling are not known. Frequent spells of allergen exposure is known to cause persistent inflammatory condition in the airway that results in tissue damage. The lungs have inherent mechanisms to handle the oxidative insult and ability to repair the tissue damage upon such insults. However, excessive and incessant oxidative insult results in the release of overwhelming amounts of inflammatory mediators, including cytokines, chemokines and growth factors. This leads to chronic inflammatory condition in the airway, which modifies the normal cell physiology that results in airway remodeling [32]. Airway remodeling involves thickening of the basement membrane due to deposition of extracellular matrix proteins, goblet cell metaplasia and mucus hyper-secretion, which result in airflow limitations leading to airway hyper-responsiveness and decline in lung function thereby compromise the structure and function of the lungs [33], [34]. In the present study, we used chronic mouse model of asthma where OVA-sensitized mice were exposed to OVA twice per week over a period of 6-weeks and examined the effect of AR inhibitor, fidarestat, administered orally starting after the first OVA-challenge, on the parameters of airway remodeling. Our results demonstrate that besides increased infiltration of inflammatory cells in the lungs and their accumulation in BAL fluid, there was an increased deposition of collagen in airways which corresponded to increased AHR in response to methacoline. Our results further demonstrate that AR inhibition significantly prevents goblet cell metaplasia caused by chronic exposure of mice to OVA. We have demonstrated earlier that AR inhibition prevents goblet cell metaplasia in the airway epithelial cells in both in-vitro in SAECs grown on air-liquid interface and in-vivo in RWE-sensitized and challenged mice lung [11]. The present results suggest stimuli -independent effect of AR inhibition in modulating mucus levels in the airways. Taken together, our in-vivo results suggest that administration of AR inhibitor to allergen-sensitized mice could prevent the airway remodeling in chronic asthmatic lungs. However, the molecular mechanisms of how inhibition of AR could prevent these events are still unclear.

The remodeling, an impaired repair mechanism, involves a number of cell types such as airway epithelial, smooth muscle, fibroblasts and immune cells and inflammatory mediators including cytokines, chemokines and growth factors released by these cells [34]. However, airway epithelial cells, which act as barrier to the external environment and control the local microenvironment and help maintain tissue homeostasis, play a crucial role in remodeling [35]. Additionally, the cross-talk between epithelial cells and the underlying mesenchymal cells is known to drive remodeling response. Out of several important mediators of remodeling, TGFβ1, released from damaged/repairing epithelium, is considered the main mediator of structural changes associated with airway remodeling. Release of TGFβ 1 in the BAL of experimental animals exposed to OVA as well as in chronic asthma patients, suggests its crucial role in the airway remodeling and lung epithelial mesenchymal transition (EMT) [36]–[38]. Therefore, to answer the questions related to the mechanism(s), we used the in-vitro cell culture model where we examined the effect of AR inhibition on TGFβ1-induced changes in SAECs and mLFs. In airway remodeling during chronic asthma, TGFβ-induced EMT of the airway epithelial cells and differentiation of myofibroblasts are recognized as primary events leading to airflow obstruction [38], [39]. In the present study, we observed that when SAECs were incubated with TGFβ1 for longer duration, a significant increase was found in the expression of EMT marker proteins such as vimentin, occludin and MMP-2. Further, in mLFs, TGFβ1 caused increased expression of fibronectin and alpha-smooth muscle actin (α-SMA). These changes in both SAECs and mLFs were significantly prevented by inhibition of AR using fidarestat. Similarly, a decreased expression of epithelial marker protein, E-cadherin, in SAECs by TGFβ1 was reverted to near normal in fidarestat-treated cells. These changes indicate that AR could modify the impaired repair process in cell culture models caused by TGFβ1.

**Figure 9:**
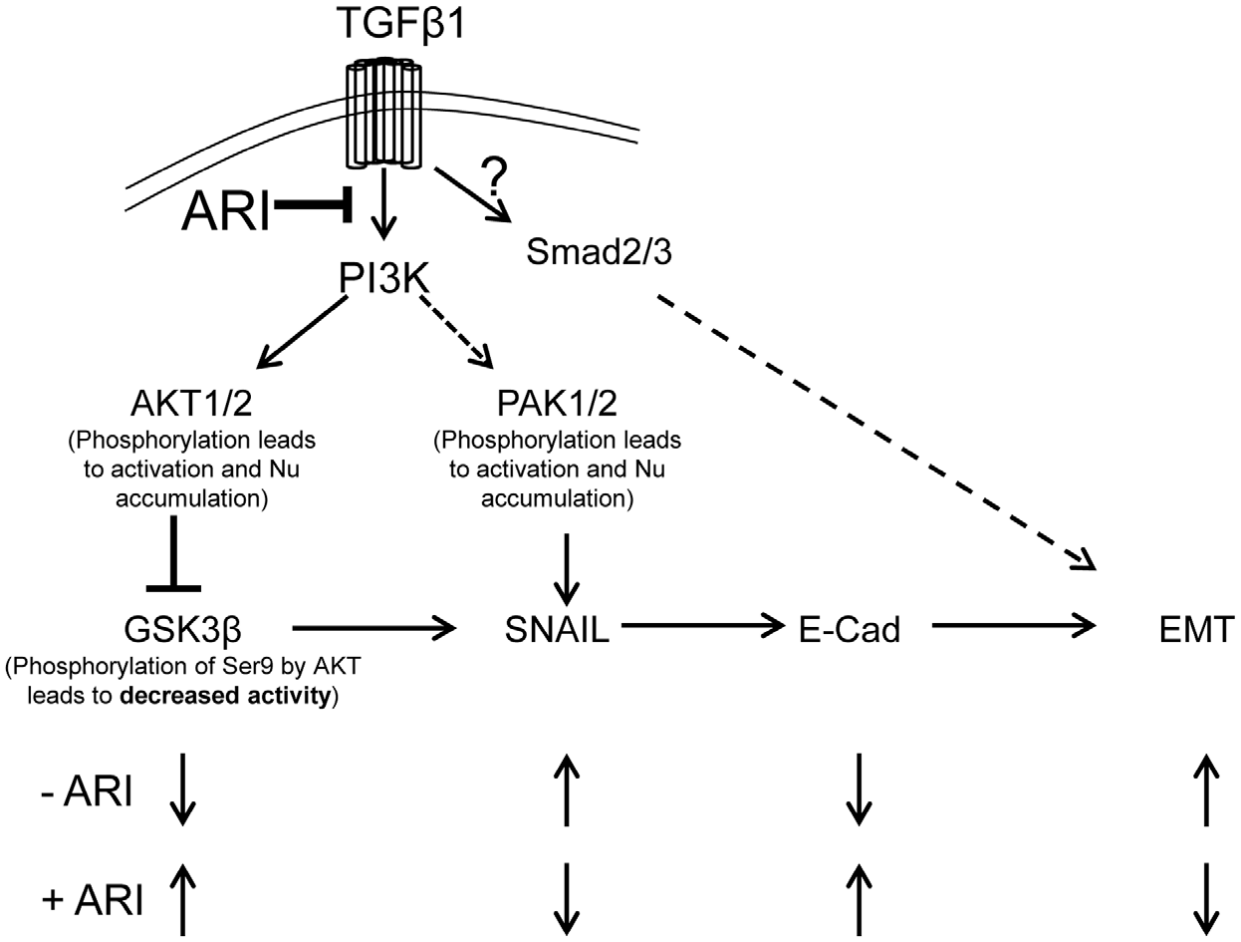
AR inhibition prevents airway remodeling in chronic asthma. Besides stimulating Smad-dependent signals, TGFβ1 is known to stimulate PI3K/AKT/GSK3β pathway which plays an important role in airway remodeling (discussed in the text). In the absence of AR inhibition, TGFβ1 activates PI3K and AKT, the later phosphorylates GSK3β resulting in its inactivation. Since GSK3β negatively regulates Snail, decreased GSK3β activity results in snail activation which then down regulates E-cadherin resulting in EMT. Based upon our results in the present study, we speculate that AR inhibition results in decreased AKT phosphorylation, which results in increased GSK3β activity, and decreased Snail activity. The later leads to increased E-cadherin levels and decreased EMT in airway epithelial cells. Thus, inhibition of AR by fidarestat significantly blocks PI3K/AKT/GSK3β pathway thereby prevents EMT related changes observed during airway remodeling. Broken lines indicate multiple steps involved.

Although, we observed that TGFβ1-induced changes in SAECs and mLFs were significantly modified by AR inhibition, when we examined canonical TGFβ1-induced pathway, AR inhibition did not significantly affect the TGFβ1-induced phosphorylation of Smad2/3, a well known effecter molecule for TGFβ1. This suggested that the effect of AR inhibition is not through Smad-dependent pathways. Even though, traditionally TGFβ1 effect is mediated via activation of Smad2/3 pathway [29], Smad-independent pathways such as phosphatidylinositol-3-kinase/protein kinase B (PI3K/AKT) and p38 MAPK have recently been proposed to actively propagate TGFβ1 signals in many cell types [40]–[45]. In the airway of asthma patients, TGFβ1 is known to activate p38 MAPK–signaling pathway which initiates apoptosis [46]. In chronic asthma patients, TGFβ1–induced p38 MAPkinase mediates airway epithelial cells apoptosis resulting in their detachment causing an injury, which along with impaired repair processes, leads to inflammatory and remodeling responses in the underlying submucosa [35]. Further, many studies have shown that PI3K gamma-deficient mice have reduced levels of allergen-induced eosinophilic inflammation and airway remodeling and do not develop airway hyper-responsiveness [47], [48]. These evidences thus suggest that PI3K could be an important player in the mediation of airway remodeling in asthma. Many investigators have suggested this possibility and shown the effectiveness of PI3K inhibition in preventing cellular changes in-vitro [49]–[51]. Therefore, we examined whether preventive effect of AR inhibition on EMT in SAEC could be via a PI3K-AKT pathway. Our results demonstrate that TGFβ1 significantly activated PI3K, AKT, GSK3β and PAK protein kinases in SAECs which were significantly prevented by inhibitors of AR, PI3K and p38. These observations suggest that the effect of AR inhibition in the prevention of remodeling and TGFβ1-induced EMT in-vitro could be through a non-canonical Smad-independent pathway. It is known that TGFβ1 phosphorylates and activates PI3K which in turn activates AKT. The active AKT then phosphorylates Ser-7 residue of GSK3β, causing its inactivation. Since GSK3β negatively regulates Snail, inactivated GSK3β results in Snail activation which would then down regulate E-cadherin resulting in EMT. In the presence of AR inhibitor, decreased phosphorylation of PI3K and AKT would result in increased GSK3β activity, decreased Snail activity and increased expression of E-cadherin (Fig. 9), which would help in maintaining the homeostasis of epithelial cells and thus prevent EMT/remodeling in SAECs.

Various studies have shown decreased airway antioxidant levels, especially reduced glutathione (GSH), in the lungs of asthmatics which correspond to the airflow limitations and are associated with lipid peroxidation byproducts including lipid aldehydes [52]–[54]. These events further increase oxidative stress in the lungs resulting in the release of cytokines, chemokines and growth factors which lead to inflammatory changes that involve aberrant growth and differentiation of airway cells such as EMT, metaplasia, hyperplasia and deposition of ECM [8], [55]. We have shown previously that AR-catalyzed reduced product of GS-lipid aldehyde, produced by oxidative stress-induced lipid peroxidation and subsequent rapid conjugation with GSH is the activator of upstream kinases including PI3K. Thus, by blocking the formation of AR-catalyzed products in the lungs, AR inhibition prevents phosphorylation of these kinases [56].

Thus, in summary, our results from the present studies demonstrate that AR mediates airway remodeling via PI3K/AKT/GSK3β-PAK pathway and that its inhibition blocks the progression of remodeling in the experimental models of chronic asthma.
